# An experimental dataset using UAVs and LoRa technology in avalanche scenarios

**DOI:** 10.1016/j.dib.2025.112243

**Published:** 2025-11-06

**Authors:** Fabio Mavilia, Davide La Rosa, Andrea Berton, Michele Girolami

**Affiliations:** a Institute of Information Science and Technologies, National Research Council (ISTI-CNR), Via G. Moruzzi, 1, 56124, Pisa, Italy; b Institute of Geosciences and Earth Resources, National Research Council (IGG-CNR), Via G. Moruzzi, 1, 56124, Pisa, Italy

**Keywords:** LoRa, Avalanche, Localization, UAV, SaR, ARTVA, ARVA

## Abstract

Wireless communication technologies play a critical role in the effectiveness of Search and Rescue (SaR) operations, especially in avalanche scenarios where rapid localization of victims is essential. Traditional systems like ARTVA research beacons have been widely adopted for this purpose, but their performance is strongly affected by environmental factors such as snow depth and snowpack characteristics. The dataset presented in this article explores the feasibility and the performance of LoRa (Long Range) technology on board of a UAV for use in SaR scenarios. The transmitter was buried in snow across a wide area in the Dolomites, simulating the scale and conditions of a typical human-triggered avalanche, while the receiver is mounted on a commercial UAV following different flight trajectories. Specifically, we vary the flying path, duration, covered area and antenna type. For each experiment, we record key communication metrics such as the Received Signal Strength Indicator (RSSI) and the Signal-to-Noise Ratio (SNR), together with precise ground truth transmitter and receiver positions obtained via GPS-RTK. The tests covered both dry and wet snow conditions, allowing evaluation of how snow characteristics impact LoRa performance.

This dataset provides strong reuse potential for researchers aiming to improve UAV-assisted localization algorithms in extreme snow environments. It can support the development and benchmarking of positioning methods based on LoRa signal strength and, more broadly, the design of resilient SaR communication systems for avalanche-prone areasBy releasing the data and contextual documentation publicly, we seek to encourage innovation in disaster response technologies and promote safer mountain rescue practices.

Specifications Table

**Table:** 

Subject	Computer Sciences
Specific subject area	Outdoor Localization; Internet of Things; UAV Localization
Type of data	csv files (dataset with Ground Truth annotation)
Data collection	Data were collected on a plateau located in the Dolomite Alps at an elevation of 1850 m, simulating an avalanche rescue scenario. A LoRa-based transmitter was buried beneath the snowpack, while a UAV equipped with a LoRa receiver conducted multiple flight trajectories to emulate a search and rescue operation. The dataset includes 19 flights, varying in flight path, antenna topology, and operating altitude. The receiver logs various signal metrics, including Received Signal Strength Indicator (RSSI) and Signal-to-Noise Ratio (SNR)
Data source location	Col de Mez, Soraga TN, Italy. GPS coordinates: 46° 22′41’’ N, 11°49′33’’ E
Data accessibility	Repository name: Zenodo Data identification number: https://doi.org/10.5281/zenodo.17339095 Direct URL to data: https://zenodo.org/records/17339095
Related research article	None

## 1 Value of the Data

• The dataset is designed to study and test outdoor localization algorithms to identify the burial location of victims of an avalanche.
• The dataset adopts a reproducible approach, detailing the simulation field, UAV paths, and snowpack type to analyze LoRa signal propagation in snow.
• UAV flights vary in duration, altitude, and covered area, simulating realistic conditions for UAV deployment in search and rescue scenarios.
• The dataset is released with an accurate Ground Truth reporting the actual UAV position obtained with GPS-RTK technology.

## 2 Background

The motivation behind compiling this dataset arises from the need to assess the feasibility and effectiveness of outdoor localization technologies in avalanche-related Search and Rescue (SaR) operations. Traditional ARTVA systems (Apparecchio di Ricerca dei Travolti in Valanga) widely adopted from winter mountaineers, while essential, face significant limitations in complex snowpack conditions and challenging terrains. To address this, the study evaluates the propagation characteristics and localization potential of wireless LoRa (Long Range) technology under controlled yet realistic field conditions. The dataset was collected following a reproducible and rigorously defined methodology to ensure consistency and scientific validity, as done in [1,2]. Specifically, the dataset includes telemetry data from Unmanned Aerial Vehicles (UAVs) whose positions were recorded using a high-precision GPS-RTK system, enabling accurate spatial referencing together with UAV telemetry, as also done in [3,4,5 and 6].

Furthermore, the UAV flight paths were varied in both duration and spatial coverage to reflect realistic aerial operations in avalanche-prone areas. These design choices ensure the dataset captures a wide range of conditions relevant to real-world SaR missions and supports robust modeling and algorithm development.

## 3 Data Description

The data is publicly available in [9] and it is organized into separate folders, one for each test performed. The types of tests conducted are described in detail in the Methods section. Each folder contains three distinct files whose structure is detailed in the following.

The first file, named *data*, is a timeseries containing RSSI and SNR values collected by the LoRa receiver mounted on the UAV. Each entry is associated with telemetry data from the drone, which serves as reference ground truth. The fields included in the timeseries are listed in Table 1 and are defined as follows:

• *dateString*: date and time indication of receiving LoRa message, according to the ISO 8601 standard.
• *timestamp*: receiving LoRa message time in epoch format.
• *rssi*: RSSI value expressed in dBm unit.
• *snr*: SNR value expressed in dB unit.
• *longitude*: longitude of the drone position expressed in WG84 reference system.
• *latitude*: latitude of the drone position expressed in WG84 reference system.
• *height*: height of the UAV above ground level [a.g.l.] in meters.
• *altitude*: UAV altitude in meters above sea level.
• *speed*: speed of the drone in meters per second.
• *depth*: the burial depth in meters of the transmitter.
• *runID*: id number indicating the run performed.

**Table 1:** Fields of the data file.

dateString	timestamp [ms]	rssi [dBm]	snr [dB]	longitude	latitude	height [m]	altitude [m a.s.l.]	speed [m/s]	depth [m]	runID

The second file, named *telemetry*, is a time series of UAV telemetry data collected during the test. Compared to the *data* file, it features a finer temporal resolution, with a granularity of tenths of a second. The fields included are listed in Table 2 and correspond directly to those defined in the *data* file.

**Table 2:** Fields of the telemetry file.

dateString	timestamp [ms]	longitude	latitude	height[m]	altitude [m a.s.l.]	speed [m/s]	runID

Additionally, the dataset contains a file, named *target_position*, which provides the precise geographical coordinates of the transmitting unit. The position data, longitude and latitude, are expressed according to the WGS84 reference system.

We include with the dataset the snowpack profile which characterize the main properties of the snow influencing LoRa radio propagation. Nivometric profiles are performed following the International Classification for Seasonal Snow on the Ground ICSIUCCS-IACS 2009 and the AINEVA Model 4 (Interregional Snow and Avalanche Association) for graphical representation. Profiles are available in the *snow profiles* folder.

## 4 Experimental Design, Materials and Methods

### 4.1 *Design*

**Fig. 1:**
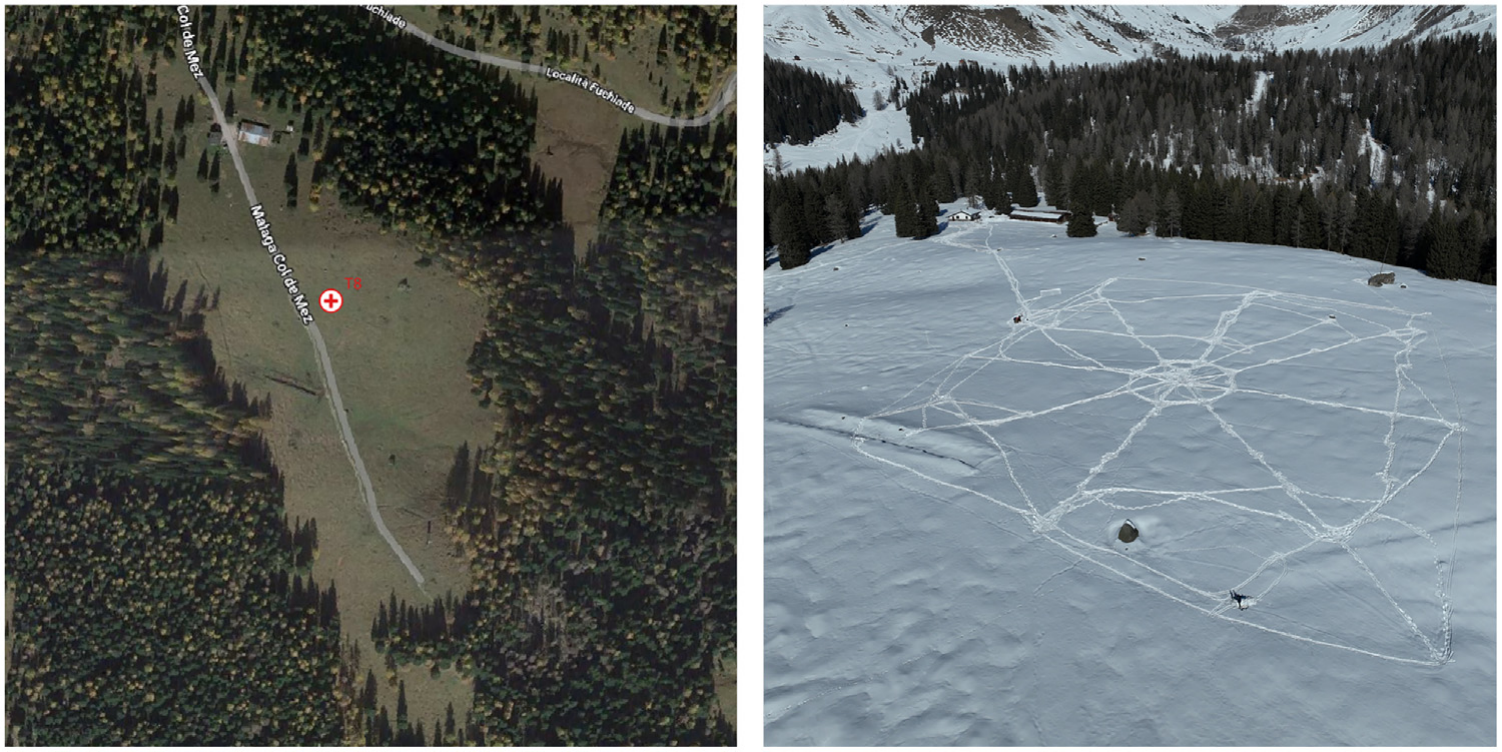
The experimental field.

The design of the dataset follows several requirements in order to provide realistic and accurate data to the research communities. Tests were conducted on a plateau at Col de Mez in the Dolomites (Soraga, TN, Italy), situated at an altitude of 1870 m above sea level and facing south. This location was chosen for its suitability for experimentation: it is a snow-covered area with easy access via a flat trail, and it experiences significant variation in snow conditions throughout the winter season. Additionally, the site is safe and well-suited for establishing a base camp. Fig. 1 shows the plateau where the tests took place. Data have been collected in the period March 2024 to April 2025. The plateau where the experiments were conducted is predominantly flat. The test area is surrounded by a dense forest of fir and larch trees with an approximate height of 15–20 m The area is free of infrastructures that could interfere with radio signal propagation, except for two protruding rocks located in the northern part of the test field. As shown in Fig. 1, a small structure is present to the north of the test area; however, it lies outside the experimental field.

The design of the data collection has been driven by set of requirements reported below:

• Reproducibility: All necessary details and environmental conditions are provided to ensure the full reproducibility of the experiments and the adopted methodology, enabling other researchers to replicate the tests under similar conditions.
• Diverse Measurement Points: Measurement points were strategically placed both near and far from the burial location, enabling the simulation of different phases of a typical SaR operation—from coarse to fine-grained localization.
• Different Operational flight: the UAV’s flights vary according to several parameters thus reproducing realistic conditions. The tested flights range from short and manual flights to automatic exhaustive survey of the testing field.
• Snowpack Variability: Data were collected during different periods of the season to capture a range of snow types and conditions.
• Snow Depth Variability: The transmitter was buried at varying depths to assess the impact of snow accumulation on signal propagation and attenuation.

### 4.2 *Material*

The dataset is obtained using LoRa boards capable of transmitting and receiving LoRa messages at 868Mhz. More specifically, we rely on the T-beam Meshtastic boards^1^ (by LILYGO) equipped with ESP32 and SX1276 chipsets. Concerning the antenna, we executed tests with two models:

• WANTENNAX019 produced by CAEN RFID^2^ with gain 8.5 dBc and right hand circularly polarized;
• GSM/GPRS Antenna L7222 by LILYGO^3^

**Fig. 2:**
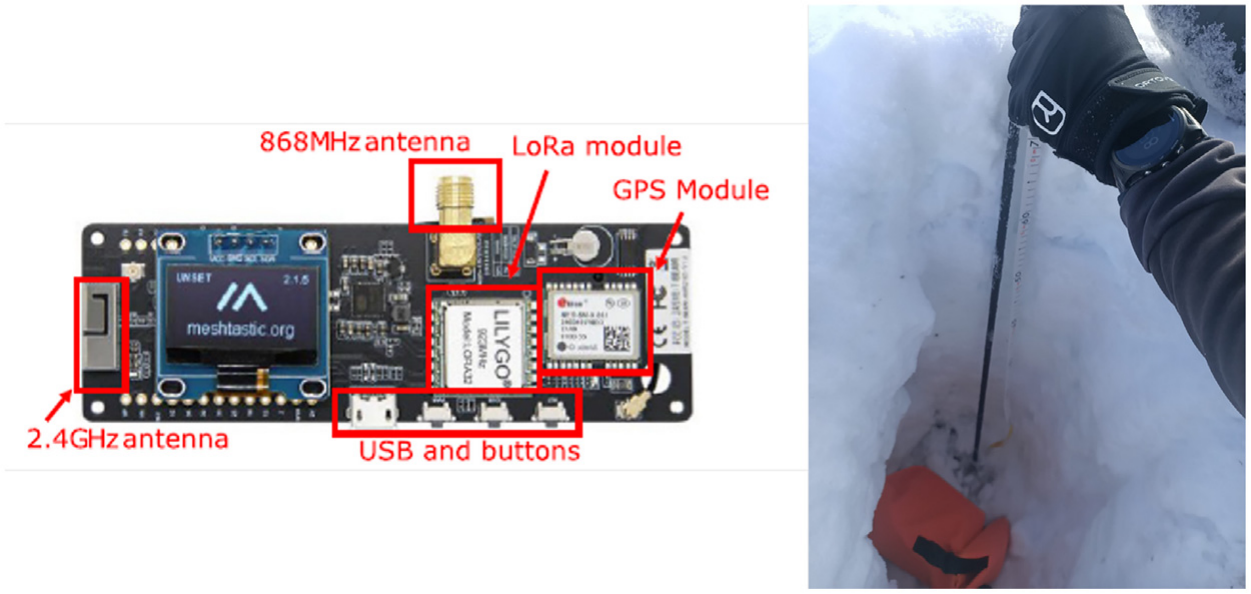
Detail of the LILYGO board and of the transmitter’s deployment.

Fig. 2 displays the LoRa module used in this study. The board incorporates a System on Chip (SoC) that combines an ESP32 processor with an SX1276 LoRa transceiver. It also includes a GPS receiver and supports both WiFi and Bluetooth connectivity. Custom Arduino-compatible firmware can be uploaded via a USB interface, leveraging 4 MB of flash memory and 8 MB of PSRAM. The transmitter is sealed in a waterproof bag and buried beneath the snowpack (Fig. 2), reproducing the “send” mode of an ARTVA (avalanche beacon).

Concerning the receiver board, it is connected to Raspberry Pi 4 units, which log packet information—such as timestamps, RSSI (Received Signal Strength Indicator), and SNR (Signal-to-Noise Ratio)—onto SD cards in text format.

**Fig. 3:**
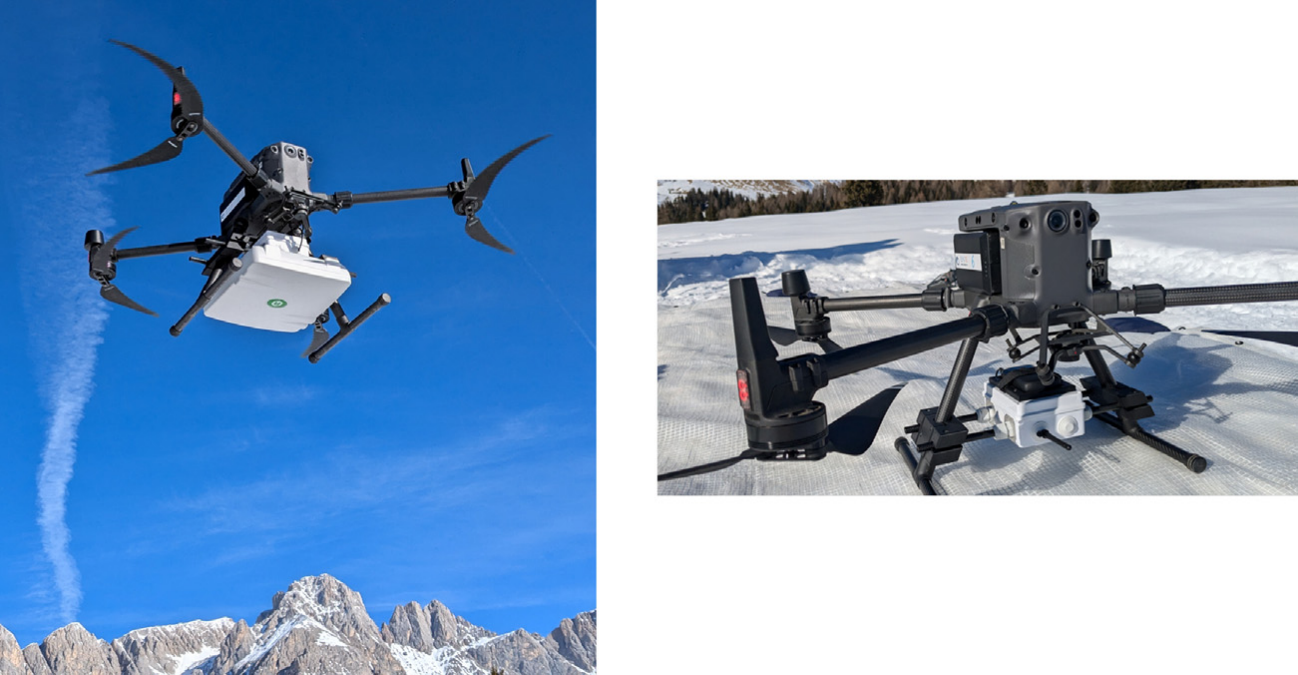
The receiver unit mounted on the UAV with an omni-directional and dipole antenna.

The receiver mounted on the UAV is time-synchronized before deployment using Wi-Fi and NTP (Network Time Protocol) to ensure accurate timestamping as shown in Fig. 3.

We deployed a custom Arduino-based firmware both on the receiver and on the transmitter reproducing a typical send-receive protocol. The adopted parameters of the LoRa module are reported in Table 3 based on some previous studies [1,7].

**Table 3:** Adopted parameters of the LoRa module.

*Setting*	*Value*
Transmission Power	14 dBm
Carrier Frequency	868 MHz (EU band)
Spreading Factor	7
Bandwidth	125 KHz
Coding Rate	4/5

The UAV used in our experiments is the DJI Matrice 300 RTK, equipped with a remote controller that records high-resolution telemetry data. This telemetry provides precise GNSS positioning and corresponding timestamps, which were utilized for accurate tracking during tests. In order to keep track of the starting and ending time of each test, we also use an Android app to record start and end times of data collection at different locations, namely the StepLogger app [8]. This app runs on a Google Pixel 6 Pro, synchronized via NTP protocol.

## *Methods*

The dataset comprises a wide variety of tests varying the flying path, altitude, duration and speed. The combination of such tests offers the possibility of studying the effectiveness of the LoRa technology for SAR operations.

Table 4 summarizes the tests we conducted, the table reports the test configurations both for the receiver mounted on board of the UAV and for the transmitter buried under the snow.

**Table 4:** Overview of tests executed during the data collection campaigns.

		UAV				Target	
ID	Date	Antenna Type	Covered Area [m^2^]	Avg. Speed [m/s]	Avg. Height [m]	Depth [m]	Antenna Polarization
T1.0	2024–04	Omnidirectional	10,000	0	15	0.5	Horizontal
T2.1	2025–02	Omnidirectional	ca. 10,000	2	20	0.4	Horizontal
T2.2		Dipole					
T3.1		Omnidirectional		2.7			
T3.2		Dipole		2			
T4.1		Omnidirectional		2.2			
T4.2		Dipole		2			
T5.1		Omnidirectional		5.4	21		
T5.2		Dipole		6.2			
T6.1	2025–04	Dipole	ca. 120,000	4.8	28.1	0.6	Horizontal
T6.2							Vertical
T7.1			ca. 70,000	4.7	35.1		Horizontal
T7.2							Vertical
T8.1			ca. 68,000	4.8	36.4		Horizontal
T8.2							Vertical
T9.1			ca. 51,000		37.1		Horizontal
T9.2							Vertical
T10.1			ca. 59,000		32		Horizontal
T10.2							Vertical

We conducted a total of 62 experiments, varying the size of the experimental field (Field 1: 10,000 m²; Field 2: up to 120,000 m²), the burial depth, and the antenna polarization, as summarized in Table 4. We believe that the diversity of the flight trajectories adequately represents a wide range of scenarios relevant to target localization tasks.

As for the flight altitude, it is essential to maintain a line of sight with the UAV while ensuring safe clearance above trees and other obstacles in the avalanche area. As reported in Table 4, we employed eight different UAV altitudes, ranging from 15 m AGL (Above Ground Level) to 37.1 m AGL. Due to the terrain morphology, flying at lower altitudes could result in collisions with trees bordering the avalanche field, whereas higher altitudes may compromise the line of sight with the UAV.

In the following, we describe each executed test.

**Fig. 4:**
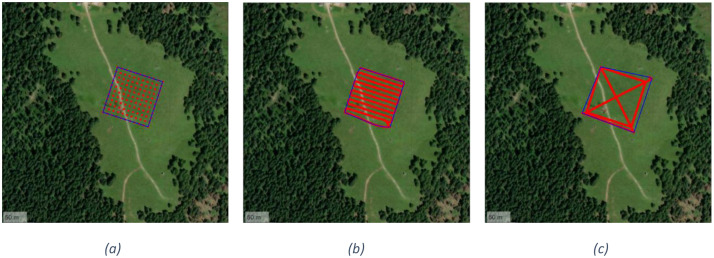
UAV flight path for tests: T1 (a), T2 (b) and T3 (c).
○ T2.1, T2.2. The UAV flies over the same 121 grid points used in the T1.0 test at a constant speed of 2 m/s, without stopping at any location. The flight altitude is maintained at 20 m The UAV is equipped with an omnidirectional antenna and a dipole antenna in T2.1 and T2.2, respectively. The followed path is shown in the Fig. 4b
○ T3.1, T3.2. The UAV performs a perimeter flight around the 100 *m* × 100 m area centered on the burial site, including both diagonals. The flight is executed automatically at a constant speed of 2 m/s and an altitude of 20 m, without any stops. The UAV is equipped with an omnidirectional antenna and a dipole antenna in T3.1 and T3.2, respectively. Fig. 4c shows the UAV flight path for tests T3.1 and T3.2.
○ T4.1, T4.2. The UAV follows a zigzag path across the entire 100 *m* × 100 m area. Specifically, it performs five diagonal transects during the north-to-south outbound flight and another five during the south-to-north return, connected by a horizontal transect. As in previous tests, the flight is conducted at a constant speed of 2 m/s and an altitude of 20 m The UAV is equipped with an omnidirectional antenna and a dipole antenna in T4.1 and T4.2, respectively. The followed path is shown in the Fig. 5a.

○ T1.0. The UAV stands at each of 121 measurement points regularly arranged in a 100 *m* × 100 m grid centered on the burial site, flying at an altitude of 15 m At each grid point, the UAV hovers for 30 s before automatically proceeding to the next location. Fig. 4a shows the grid of 121 measuring points above the Col de Mez plateau. The rationale of this test is providing an accurate evaluation of the LoRa signal propagation with an extended number of measuring points.

**Fig. 5:**
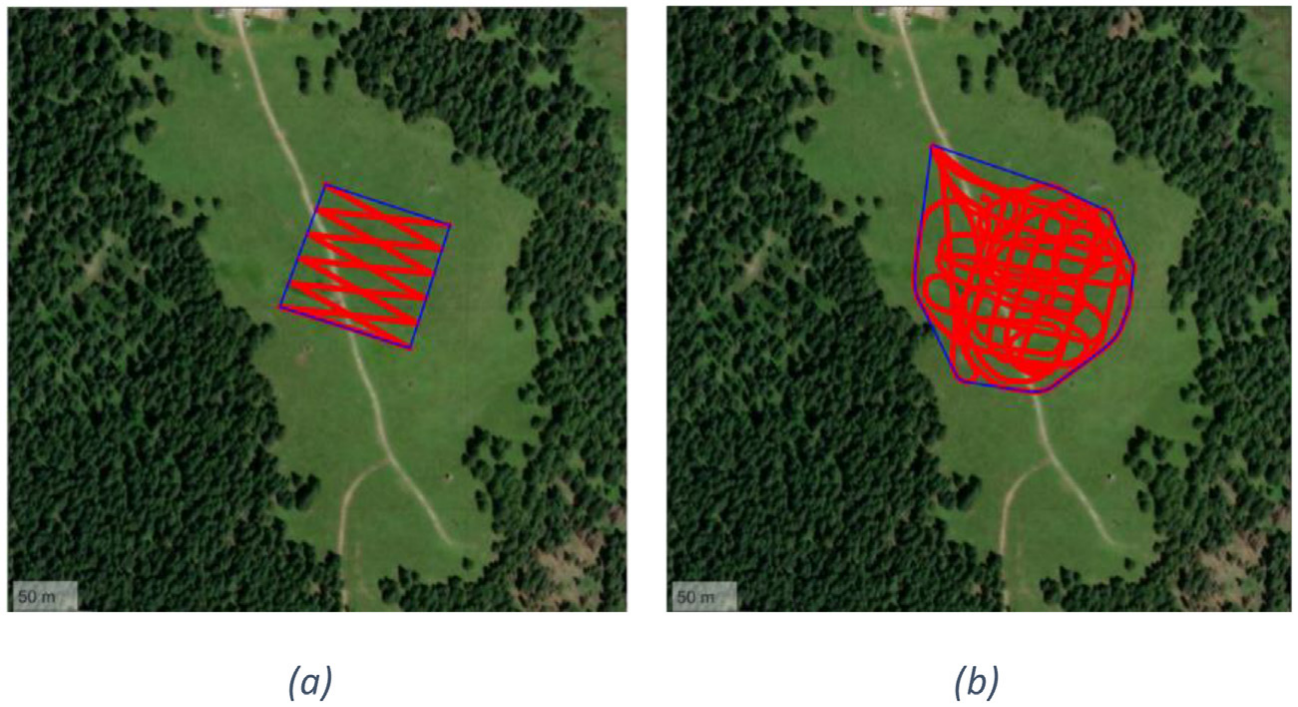
Overview of UAV flight paths for T4 (a) and T5.1 (b).
○ T5.1, T5.2. The UAV follows a random trajectory manually controlled by the pilot within the 100 *m* × 100 m area. In contrast to previous tests, the flight path is not predetermined. The flight altitude is set to 21 m, with average overflight speeds of 5.4 m/s and 6.2 m/s for tests T5.1 (omnidirectional antenna) and T5.2 (dipole antenna), respectively. Fig. 5b shows the single run performed for test T5.1, while Fig. 6 details the three different paths followed by the UAV for the three different runs of test T5.2. The rationale behind these tests is reproducing the UAV path controlled by a ground operator during a realistic SAR operation, where it is not possible to exactly define the boundaries of the avalanche field.

**Fig. 6:**
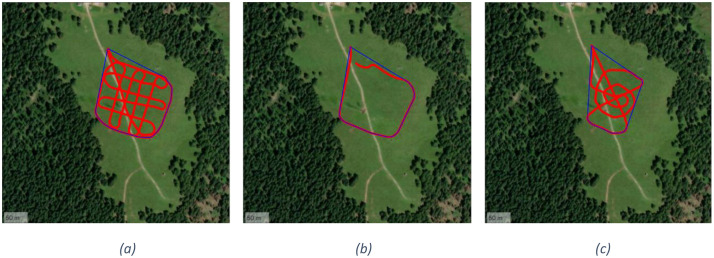
Random trajectories manually controlled by the pilot for all three runs of test T5.2: run 1 (a), run 2 (b) and run 3 (c).
○ T6.1, T6.2. The UAV follows a significantly wider flight path compared to previous tests, covering beyond the entire Col del Mez plateau, an area exceeding 100,000 square meters. In tests T6.1 and T6.2, the UAV performs a perimeter flight of the area, including both diagonals. The flight path is illustrated in Figure 7Figure 6a. To avoid tree obstacles, the flight altitude is increased to 28 m, and the average speed is set to 4.8 m/s. The transmitter antenna is configured with horizontal polarization for test T6.1 and vertical polarization for test T6.2. The rationale of these tests is to remarkably extend the research area to stress the receiving unit on board of the UAV. With such settings, we experienced a maximum distance between transmitter and receiver of approximately 320 m.
○ T7.1, T7.2. The UAV follows a zigzag path across the entire plateau, covering an area of ca. 70,000 square meters. The flight is executed automatically at a constant speed of 4.7 m/s and an altitude of 35 m, without any stops. The transmitter antenna is configured with horizontal and vertical polarization for tests T7.1 and T7.2, respectively. Fig. 7b shows the UAV flight path.

**Fig. 7:**
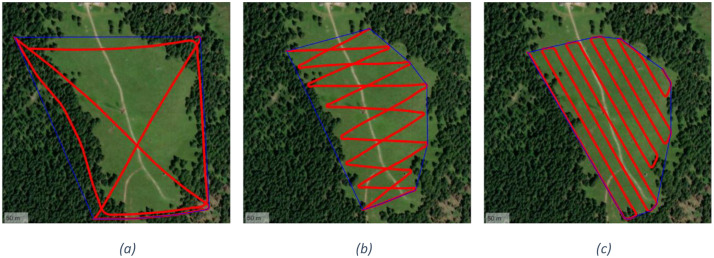
UAV flight path for tests: T6 (a), T7 (b) and T8 (c).
○ T8.1, T8.2, T9.1, T9.2. The UAV follows a predefined flight pattern known as the *Greek key*, consisting of a vertical line along the longer side of the area followed by a short horizontal transect. In tests T8.1 and T8.2, the UAV covers the entire plateau using 10 vertical lines and 9 horizontal transects (Fig. 7c). In contrast, in tests T9.1 and T9.2, the path is less dense, consisting of 4 vertical lines and 3 horizontal transects (Fig. 8a). The flight is executed automatically at a speed of 4.8 m/s and an altitude of 36.4 – 37.1 m, without any stops. The transmitter antenna is configured with horizontal and vertical polarization for tests T8.1/T9.1 and T8.2/T9.2, respectively.

**Fig. 8:**
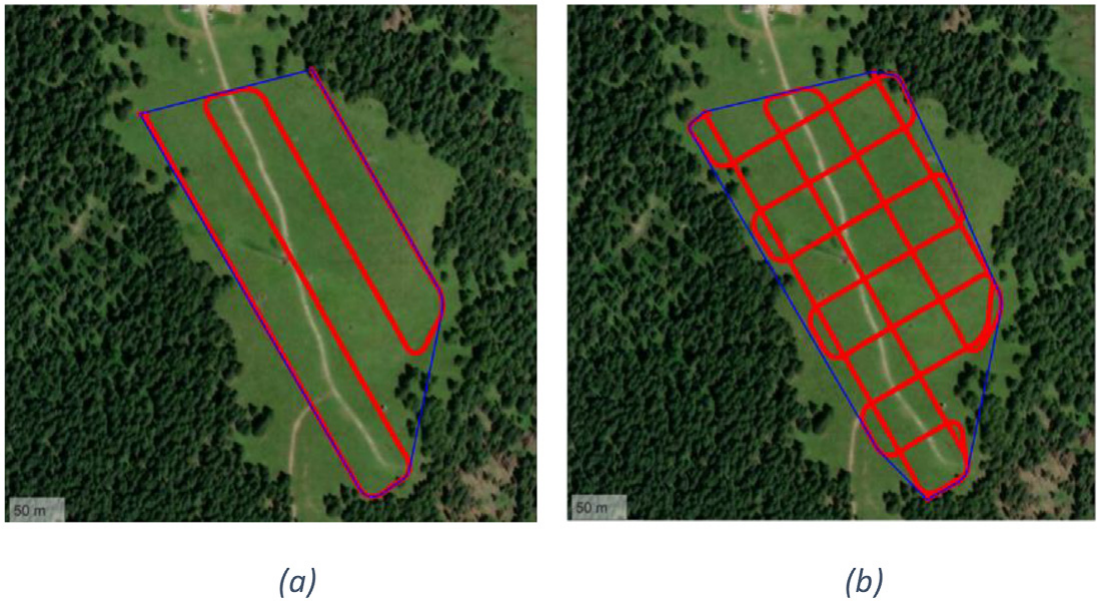
Overview of UAV flight paths for T9 (a) and T10 (b).
○ T10.1, T10.2. In the final test, the UAV executes a double cross-pattern, forming a grid that covers the entire plateau area. The path is shown in the Fig. 8b The speed remains constant at 4.8 m/s, at an altitude of 32 m The transmitter antenna is configured with horizontal polarization for test T10.1 and vertical polarization for test T10.2.

Table 5 provides details concerning the amount of the collected values (number of LoRa messages successfully received by the UAV) for each test and the duration. Overall, the dataset consists of over 140,000 samples, collected over a total duration exceeding 8 h.

**Table 5:** Quantitative Summary of the Dataset.

ID	Performed Runs	Collected Values	Duration [min.]
T1.0	1	16,624	69.39
T2.1	3	11,458	40.55
T2.2		8294	29.48
T3.1		4846	17.18
T3.2		5793	20.60
T4.1		9218	32.72
T4.2		8304	29.58
T5.1	1	3821	13.55
T5.2	3	2616	9.28
T6.1	3	7188	26.18
T6.2	2	4903	17.46
T7.1	3	8118	28.96
T7.2		8125	28.86
T8.1		8832	31.23
T8.2		8803	31.33
T9.1		4150	14.68
T9.2		4099	14.57
T10.1		8424	29.74
T10.2		8423	29.72
Total		142,039	515,06

## Limitations

The data is publicly available in [9] and it is the result of a series of data collection campaigns carried out between March 2024 and April 2025. This extended timeframe allows for testing under a wide range of snow conditions—for example, from dry to very wet snow. However, the current version of the dataset includes tests conducted under two primary conditions: nearly dry and wet snow. We plan to expand the dataset by incorporating additional snow conditions to enhance its overall representativeness and applicability.

## Ethics Statement

Authors have read and follow the ethical requirements for publication in Data in Brief and confirming that the current work does not involve human subjects, animal experiments, or any data collected from social media platforms.

## Credit Author Statement

**Fabio Mavilia:** Conceptualization, Methodology, Investigation, Visualization.

**Davide La Rosa:** Methodology, Investigation, Visualization.

**Andrea Berton**: Conceptualization, Methodology, Investigation, Visualization.

**Michele Girolami:** Conceptualization, Methodology, Supervision, Investigation, Visualization.

## Data Availability

ZenodoAn Experimental Dataset Using UAVs and LoRa Technology in Avalanche Scenarios (Original data).

## Footnotes

2 https://www.caenrfid.com/it/products/wantennax019/.

3 https://www.lilygo.cc/products/gsm-gprs-antenna.

## Footnotes

1 https://www.lilygo.cc/products/t-beam-v1-1-esp32-lora-module.
